# Serological, fragmentomic, and epigenetic characteristics of cell-free DNA in patients with lupus nephritis

**DOI:** 10.3389/fimmu.2022.1001690

**Published:** 2022-12-12

**Authors:** Fang Wang, Hai-bing Miao, Zhi-hua Pei, Zhen Chen

**Affiliations:** ^1^ Department of Rheumatology and Immunology, The Second Affiliated Hospital of Fujian Medical University, Quanzhou, Fujian, China; ^2^ Department of Immunology, Foresea Life Insurance Guangxi Hospital, Nanning, Guangxi, China; ^3^ Hubei Provincial Key Laboratory of Agricultural Bioinformatics, College of Informatics, Huazhong Agricultural University, Wuhan, Hubei, China

**Keywords:** circulating cell-free DNA, lupus nephritis, serology, fragment omics, epigenetics, liquid biopsy

## Abstract

**Objectives:**

The biological characteristics of plasma circulating cell-free DNA (cfDNA) are related to the pathogenesis of lupus nephritis (LN). The aim of this study was to explore the biological characteristics of cfDNA in patients with LN in terms of serology, fragment omics, and epigenetics, and to discuss the possibility of liquid biopsy for cfDNA as an alternative to conventional tissue biopsy.

**Methods:**

cfDNA was extracted from plasma samples of 127 patients with systemic lupus erythematosus (64 with LN, 63 without LN). The cfDNA concentration was determined using the Qubit method. Next-generation sequencing cfDNA methylation profiling was performed for three LN patients and six non-LN patients. The methylation panel was designed based on data from The Cancer Genome Atlas cohort. The fragmentation index, motif score, and DELFI score were calculated to explore the fragmentation profile of cfDNA in patients with LN. Statistical and machine learning methods were used to select features to calculate the methylation scores of the samples.

**Results:**

Patients with LN had significantly lower cfDNA concentrations (P = 0.0347) than those without LN. This may be associated with the presence of anti-double-stranded DNA antibodies (r = –0.4189; P = 0.0296). The mean DELFI score (proportion of short fragments of cfDNA) in patients with LN was significantly higher than that in patients without LN (P = 0.0238). Based on the pan-cancer data, 73, 66, 8, and 10 features were selected and used to calculate the methylation scores. The mean methylation scores of these features in patients with LN differed significantly from those in patients without LN (P = 0.0238).

**Conclusions:**

The specificity of cfDNA in patients with LN was identified using serological, fragmentomic, and epigenetic analyses. The findings may have implications for the development of new molecular markers of LN.

## Introduction

Systemic lupus erythematosus (SLE) is a multisystem autoimmune disease that can cause chronic tissue inflammation and damage. Although SLE has both environmental and genetic components, the etiology of the disease is not fully understood. Recent genetic association studies have identified more than 20 SLE susceptibility loci (1). Odds ratios for these associations have been modest, and one of the potential influencing factors may be the extensive clinical heterogeneity of SLE. In addition, an increasing amount of evidence has highlighted the contribution of epigenetic mechanisms to SLE (2, 3).

Lupus nephritis (LN) is the leading cause of end-stage renal disease and death in SLE patients (4, 5). The absence of sensitive tests to monitor renal involvement ultimately results in high mortality, high hospitalization rates, and a heavy economic burden. The highly variable clinical manifestations present a challenge for clinical management of the disease. Autoantibodies directed against native double-stranded DNA (dsDNA) are observed in 40–60% of SLE patients. Anti-dsDNA autoantibodies can be present before the clinical symptoms of SLE and are implicated in the pathogenesis of LN (6). The prevailing idea is that immune complexes activate complement factors and Fc gamma receptor-bearing cells to initiate pathological inflammatory responses.

Cell death results in the release of antigens such as nucleic acids, which combine with antibodies to form immune complexes. The complexes trigger a series of immune responses against the tissue, especially in the renal tissue of SLE patients (7). Circulating cell-free DNA (cfDNA), a type of extracellular DNA, is thought to be released into the bloodstream by necrotic cells (8). Many studies have found that cfDNA concentrations are significantly elevated in patients with SLE, especially in those with severe disease (9–14). The circulating DNA that forms immune complexes with autoantibodies in SLE patients displays a characteristic fragmentation pattern (15). Hypomethylated DNA may be potentially pathogenic for SLE owing to its stronger affinity for antibodies (14, 16). Research on cfDNA in SLE has evolved, with initial studies mainly focusing on the detection and quantification of cfDNA, followed by studies to associate cfDNA levels with disease activity, progression, and/or monitoring treatment response. However, very few studies have reported the detailed biological characteristics of cfDNA in LN.

Some studies (17, 18) attempted to explore the characteristics of cfDNA in patients with LN. However, no definitive conclusions were reached. In this study, the biological characteristics of cfDNA in the plasma of patients with LN are described in detail in terms of serology, fragment omics, and epigenetics.

## Methods

### Study participants

All patients fulfilled the 2019 American College of Rheumatology/European League Against Rheumatism SLE classification criteria (19). The exclusion criteria were gestational or lactating women and the presence of other diseases (such as other autoimmune diseases, acute or chronic infections, and tumours). Any of the following criteria were required for an LN diagnosis (1): a 24-hour quantitative urine protein level of >0.5 g or a urine albumin/creatinine ratio >500 mg/g (50 mg/mmol) (2), active urine sediments (e.g., white cell count of >5/high-power field and exclude urinary infection or red blood cell count of >5/high-power field), and (3) evidence of one or more lesions on a renal biopsy based on the International Society of Nephrology/Renal Pathology Society 2003 classification criteria (20). This study was approved by the Ethics Committee of the Second Affiliated Hospital of Fujian Medical University and was conducted in accordance with the ethical standards of the 1964 Helsinki declaration and its later amendments. All participants provided written informed consent.

### Clinical assessments and data collection

Eligible participant data were collected at the time of blood collection. Clinical laboratory examinations were performed at the Department of Laboratory Medicine of the Second Affiliated Hospital of Fujian Medical University. SLE disease activity was evaluated using the SLE disease activity index (SLEDAI) (21). Active SLE was defined as a SLEDAI score of >4. The collected data included (1) demographic data: gender, age, and body mass index; (2) inflammatory indicators: neutrophil count, neutrophilic granulocyte percentage, C-reactive protein level, and erythrocyte sedimentation rate; (3) renal function markers: albumin, serum creatinine, estimated glomerular filtration rate, urinary red blood cell, and 24-hour urine protein levels; (4) immune function indicators: complement 3, complement 4, and immunoglobulin A, G, and M levels; (5) SLE auto-antibodies: anti-U1 ribonucleoprotein, dsDNA, -Smith, -ribosomal ribonucleoprotein, -Sjogren syndrome A, and -Sjogren syndrome B; (6) immunosuppressive therapy: corticosteroids, antimalarials, methotrexate, cyclophosphamide, tacrolimus, mycophenolate mofetils, and cyclosporin A; and (7) the SLEDAI score.

### Sample preparation

Peripheral blood samples were collected in ethylenediaminetetraacetic acid-containing tubes and processed within 4 h of venipuncture. Approximately 10 mL of blood was centrifuged at 1600 × g for 10 min at 4°C. The supernatants were transferred into new microtubes and centrifuged again at 16 000 × g for 10 min at 4°C to completely remove the cell debris. The plasma samples were collected and immediately stored at −80°C until use.

### Extraction and quantification of plasma cfDNA

The MagMAX Cell-Free DNA Isolation Kit (Life Technologies, Carlsbad, CA, USA) was used to isolate cfDNA from 3-mL plasma samples. For each sample, the extracted cfDNA concentration was quantified using a Qubit dsDNA HS Assay Kit (Life Technologies, Carlsbad, CA, USA). All protocols were performed according to the manufacturer’s instructions.

### Fragmentation index of cfDNA

For quality control of the cfDNA concentration, microfluidic on-chip electrophoresis was performed using an Agilent 2100 Bioanalyzer (Agilent Technologies, Santa Clara, CA, USA) with high-sensitivity DNA chips. The number of electropherogram peaks was counted, each representing a set of equal-sized DNA fragments detected in the sample. The manufacturer’s specification stipulated a sizing resolution of 10% for 50-600 bp fragments and 20% for 600-7000 bp fragments. Thus, the cfDNA fragment lengths varying<10% and 20%, respectively, were considered the same length. The number of recalculated peaks representing different cfDNA lengths was referred to as the fragmentation index.

### cfDNA methylation sequencing

To ensure the generation of sequencing libraries, each sample contained at least 20 ng of DNA. Use of as much DNA as possible was preferred. Based on this criterion, 12 patients were included in the cohort. To ensure sequencing quality, a more stringent inclusion criterion was imposed (>40 ng DNA in each sample). Nine samples were included according to these stringent criteria. There were three cases in the LN group and six cases in the non-LN group. All nine cases, all of which had a SLEDAI score >4. No significant differences in patient demographics (age, body mass index, gender) were detected between the two groups (**Supplementary Table 1**). Logistic multi-factor regression analysis showed that demographic factors had no influence on cfDNA. cfDNA methylation sequencing was conducted at GeneCast Biotechnology Co., Ltd. (Beijing, China) based on a 1 Mb capture panel designed to cover differentially methylated regions (DMRs) merged from differentially methylated positions (DMPs) selected based on a 450k array publicly available The Cancer Genome Atlas (TCGA) data (**Supplementary Table 2**). The extracted cfDNA was used to generate WMS libraries with the NEBNext Enzymatic Methyl-seq Kit (New England Biolabs, Ipswich, MA, USA) according to the manufacturer’s instructions. Libraries were amplified using nine cycles of polymerase chain reaction and quantified using the Qubit dsDNA HS Assay Kit (Life Technologies, Carlsbad, CA, USA). The final sequencing libraries were sequenced on a NovaSeq 6000 (Illumina, San Diego, CA, USA) with a read length of paired-end 151 bp.

### Methylation sequencing data processing

Methylation sequencing reads were demultiplexed by Illumina bcl2fastq and adaptors were trimmed by Trimmomatic (v.0.36). Reads were aligned against the human reference genome (version hg19) and deduplicated by BisMark. Samtools (v.1.3) and BamUtil (1.0.14) were used for mapped reads sorting and overlap-clipping. Reads with mapping quality below 20 were filtered out before analysis.

### Calculation of motif score

The Motif score was the frequency of each plasma DNA end motif [the first 4-nucleotide (i.e. 4-mer) sequence on each 5’ fragment end of plasma DNA] (22). The frequency of plasma DNA motif CCCA was calculated *via* the following steps: i) extracting unique mapped reads with CCCA motif at 5’ end from the aligned bam files; ii) Filtering the reads with a quality value lower than 30; iii) calculating the ratio of the CCCA motif reads number and the total reads number as end motif frequency (22).

### Calculation of DELFI score

According to the research of Cristiano, S. et al. (23), the DELFI score (DNA evaluation of fragments for early interception) was calculated *via* the corrected ratio of coverage of short (100–150 bp) and long (151-220 bp) fragments within each targeted region, in which the ration was corrected by genome GC content using LOESS-based method.

### Selection of DMRs in renal carcinoma

We selected the DMPs for renal carcinoma using the array data of primary tumors and normal tissues from TCGA KIRC, KIRP, and KICH cohorts and the data of peripheral blood from GSE40279. 5,000 tissue-specific DMPs with the largest mean beta-value differences and FDRs less than 0.05 between tumor and blood samples were selected for each cohort based on their beta-values. DMPs with mean beta-value differences larger than 0.3 and FDRs less than 0.05 were selected from KIRC, KIRP and KICH individually. FDRs were calculated from the moderate t-test p-value obtained by Limma package. Among each DMP set, DMPs with distances less than 250 bp from each other were merged into a DMR (**Supplementary Table 3**) (24).

### Selection of MCBs in pan-cancer panel

Methylation-correlated blocks (MCBs) were defined as regions covering CpGs with highly-correlated beta-values (25). cfDNA Samples were hybridized into a self-designed methylation panel covering about 1Mb hypermethylated regions and 82,400 CpGs. Correlations between each pair of neighboring CpG sites were calculated in 195 formalin-fixed paraffin-embedding samples (pan-cancer except for kidney cancer) from GeneCast Biotechnology Co. Ltd. Adjacent CpG sites covered by the 1Mb panel were merged into MCBs based on the correlations of beta-values (**Supplementary Table 4**). An MCB was defined as a genomic region that contains at least 3 CpG sites, each site with< = 100 bp distance and > = 0.95 Pearson’s correlation coefficient to its adjacent site. Only those blocks with high correlations were considered.

### Measurement of methylation score

Features were selected in the cohort based on the DMRs or MCBs by using the Limma package and under the condition of *P*-value< 0.05 and *P*-value< 0.01 respectively. Two different methods were used to measure the methylation level of the selected features. One was the mean beta-value, which was the average of the beta-values of CpGs on a region. The other one was the methylated fragment ratio (MFR). MFR was defined as the fraction of fragments with fully methylated status on a region. To calculate MFR, read pairs were merged into fragments, and those failing to meet the following criteria were filtered out before analysis: i) covering at least 3 CpG sites in the region; ii) with a conversion rate no less than 95%. Using the cfDNA samples in the non-LN group as the baseline samples, we first measured the difference of each feature between the test cfDNA and the baseline MFR distribution by a Z-score, which was calculated by


$$Z_{i}=\frac{\mu_{i}}{\sigma_{i}}$$

where *μ*_*i*_ and *σ*_*i*_ were the mean and the standard deviation of MFR of the *i* th MCB feature among baseline samples, respectively.

{*Z*_1_,*Z*_2_,…, *Z*_*I*_} of the MCB feature set *I* were transformed to p-values {*p*_1_,*p*_2_,…, *p*_*I*_} and combined into a methylation score according to Fisher’s method (26).


$$\frac{\sum_{i=1}^{I}-2c_{i}\ln p_{i}}{\sum_{i=1}^{I}c_{i}}$$

where *c*_*i*_ is the total number of fragments on the *i* th MCB.

### Machine learning models

In order to prevent over-fitting caused by the excessive number of features, the features were modeled by machine learning. The machine learning models were built using the Scikit-learn package. Logistic regression, decision tree classifier, random forest classifier, and SVC were used for modeling, and the leave-one-out method was used for cross-validation. Decision tree classifier: the quality of a split was measured by entropy. Random forest: the number of trees was set to 30. Other hyperparameters were set by default. We did not tune the hyperparameters because of the small sample size to avoid the potential overfitting problems brought by the hyperparameter tuning process. The input variables were shown in **Supplementary Table 5**.

### Statistical analyses

Based on the degree of similarity with the normal distribution, the patient characteristics were expressed as the mean (standard deviation) or median (interquartile range). Absolute and relative frequencies were reported for the categorical variables. The Student’s t-test and the Mann-Whitney U test were used to analyse differences between two groups of normally and non-normally distributed variables, respectively. The data of cfDNA methylation sequencing were analyzed by Wilcoxon rank sum test. The chi-square test was used to compare categorical variables. The Spearman’s rank correlation test was used to analyse the correlation between two non-normally distributed variables. All analyses were performed using the SPSS V. 26.0 (IBM Corp., Armonk, NY, USA), and GraphPad Prism V.8.0 (GraphPad Software Inc., San Diego, CA, USA) was used to produce the graphs. The calculations were based on 95% confidence intervals. The significance level was set at a two-sided *P*-value of<0.05.

## Results

### Low concentrations of plasma cfDNA in patients with LN

In total, 127 patients with SLE were enrolled. Of these, 64 had LN and 63 did not. **Table 1** presents the patients’ demographic and clinical characteristics. Patients with LN had a higher body mass index (*P* = 0.028) and lower immunoglobulin G level (*P* = 0.02). The blood and urine indices related to renal function were all significantly abnormal in LN patients, including albumin (*P* = 0.001), serum creatinine (*P* = 0.009), urinary red blood cells (*P<* 0.001), and 24-hour urinary protein levels (*P<*0.001). The proportion of patients with anti-dsDNA antibodies was higher (*P* = 0.005) and the proportion of patients with anti-Sjogren’s syndrome A antibodies was lower (*P* = 0.026) in the LN group than in the non-LN group. Patients with LN were more often treated with cyclophosphamide (*P<* 0.001) and tacrolimus (*P* = 0.007). There was no significant bias in the choice of other drugs. SLEDAI scores were significantly higher in the LN group than in the non-LN group (*P<* 0.001). In the LN group, 82.8% of patients (n = 53) had an SLEDAI score >4, and 55.6% of patients (n = 35) in the non-LN group had a score >4 (*P* = 0.001).

**Table 1:** Patient demographics and clinical characteristics.

	LN (n=64)	Non-LN (n=63)			*P*-Value
Age,years	34.29 ± 11.67	30.93 ± 11.16			NS
Gender,female,n(%)	51(79.69)	56(88.89)			NS
BMI, kg/m^2^	21.09(19.61-23.94)	18.95(17.63-22.52)			0.028
NEUT,×10^9^/L	4.57(2.81-7.6)	3.4(2.58-4.42)			NS
NEUT%	64.91 ± 11.07	63.71 ± 12.73			NS
CRP,mg/L	2.52(1.22-4.89)	2.14(0.64-4.71)			NS
ESR,mm/H	35.5(19.75-60.25)	30.5(16-52)			NS
Alb,g/L	32 ± 7.95	39.34 ± 8.66			0.001
SCr,umol/L	65(49.2-89.7)	54(46.53-62.75)			0.009
eGFR,ml/min/1.73m^2^	50.51(45.83-56.76)	53.73(50.34-57.94)			NS
URBC,/HPF	5(2.9-8.7)	1.7(0.13-2.55)			< 0.001
UP,mg/24h	1990(720-3222)	85.5(50.25-139.25)			< 0.001
C3 level,g/L	0.558 ± 0.25	0.662 ± 0.224			NS
C4 level,g/L	0.1308 ± 0.0895	0.137 ± 0.0751			NS
IgA level,g/L	2.86 ± 1.45	2.58 ± 1.5			NS
IgG level,g/L	16.1(12.25-17.45)	18.65(15.98-23.15)			0.02
IgM level,g/L	1.05(0.63-1.59)	1.18(0.83-1.53)			NS
Anti-dsDNA,positive,n(%)	35(54.69)	19(30.16)			0.005
Anti-U1RNP,positive,n (%)	33(51.56)	35(55.55)			NS
Anti-Sm,positive,n(%)	25(39.06)	24(38.1)			NS
Anti-rRNP,positive,n(%)	29(45.31)	29(46.03)			NS
Anti-SSA,positive,n(%)	28(43.75)	40(63.39)			0.026
Anti-SSB,positive,n(%)	8(12.5)	15(23.81)			NS
**Corticosteroids, n(%)**	64(100)	63(100)			NS
**Antimalarials, n(%)**	61(95.31)	61(96.83)			NS
**Methotrexate, n(%)**	0(0)	6(9.52)			NS
**Cyclophosphamide, n(%)**	27(42.11)	2(3.17)			< 0.001
**Tacrolimus, n(%)**	13(20.31)	0(0)			0.007
**Mycophenolate mofetils, n(%)**	17(26.56)	8(12.7)			NS
**Cyclosporine A, n(%)**	0(0)	10(15.87)			NS
SLEDAI score	12(8-22)	6.5(4-9.75)			< 0.001
Active SLE(SLEDAI>4), n(%)	53(82.8)	35(55.6)			0.001

n(%), number of patients (percent); NS, not significant; LN, lupus nephritis; BMI, body mass index; NEUT, neutrophil count; NEUT%, neutrophilic granulocyte percentage; CRP, C-reactive protein level; ESR, erythrocyte sedimentation rate; Alb, albumin; SCr, Serum creatinine; eGFR, estimated glomerular filtration rate; URBC, urinary red blood cells; HPF, high power field; UP, 24-hour urinary protein; C, complement; Ig, immunoglobulin; dsDNA, double-stranded DNA; U1RNP, U1 ribonucleoprotein; Sm, Smith; rRNP, ribosomal ribonucleoprotein; SSA, Sjogren syndrome A; SSB, Sjogren syndrome B; SLE, systemic lupus erythematosus; SLEDAI, SLE disease activity index.

**Figure 1:**
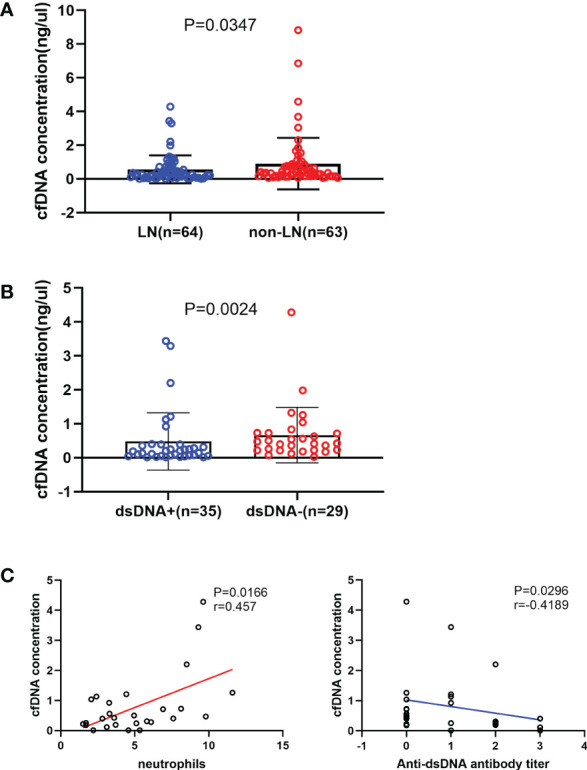
Relationship between the cell-free DNA (cfDNA) concentration and the severity of systemic lupus erythematosus. **(A)** The cfDNA concentration was significantly lower in the LN (n = 64) group than in the non-LN (n = 63) group (0.257 [0.107-0.61] ng/µL vs 0.406 [0.178-0.818] ng/µL; *P* = 0.035). **(B)** LN patients with anti-double-stranded (ds) DNA antibodies (n = 35) had a significantly lower cfDNA concentration than those without anti-dsDNA antibodies (n = 29; 0.188 [0.051-0.393] ng/μL vs 0.422 [0.218-0.731] ng/μL, *P*=0.0024). **(C)** The cfDNA concentration increased with increased neutrophil counts (*r*=0.457, *P*=0.0166) and decreased with increased anti-dsDNA antibodies titre in LN group (*r*=-0.4189, *P*=0.0296).

The LN group had a significantly lower cfDNA concentration than the non-LN group (*P* = 0.0347; **Figure 1A**). The production of autoantibodies in serum is a characteristic of SLE. Patients with LN were grouped according to the presence or absence of anti-dsDNA antibody. cfDNA concentration was significantly lower in patients with anti-dsDNA antibody than in those without anti-dsDNA antibody (*P* = 0.0024; **Figure 1B**). The correlation between cfDNA concentration and clinical indicators was analyzed in patients with LN. cfDNA concentration was positively correlated with neutrophil count (*r* = 0.457; *P* = 0.0166) and negatively correlated with anti-dsDNA antibody titer (*r* =–0.4189; *P* = 0.0296) (**Figure 1C**). There was no correlation between cfDNA concentration and other clinical indicators in LN patients, such as neutrophilic granulocyte percentage, C-reactive protein, erythrocyte sedimentation rate, albumin, serum creatinine, estimated glomerular filtration rate, urinary red blood cells, 24-h urinary protein, complement 3 or complement 4, immunoglobin, and SLEDAI score (*P* > 0.05). cfDNA concentrations were also unaffected by treatment with cyclophosphamide or tacrolimus (*P* > 0.05).

### Short fragments of plasma cfDNA in patients with LN

**Figure 2:**
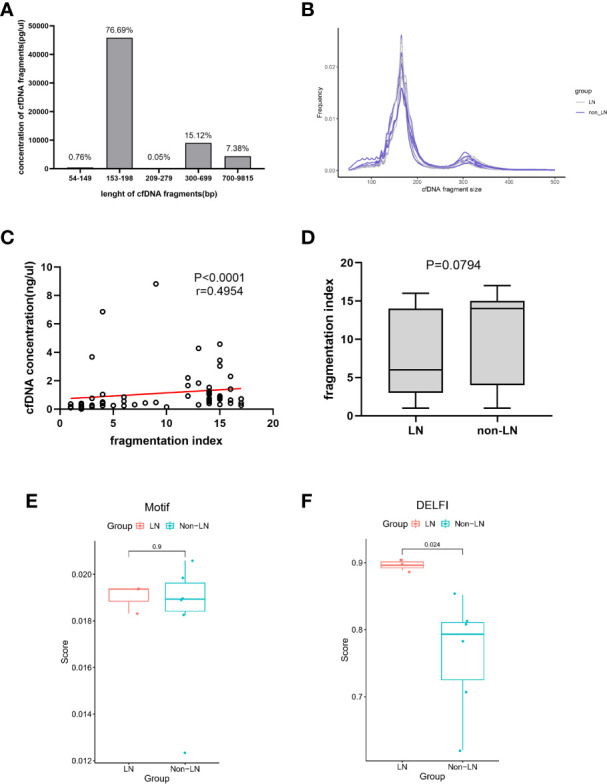
The fragmentation profile of plasma cfDNA. **(A)** The proportion of cfDNA of different fragment lengths. Data was generated using a High Sensitivity DNA chip on the Agilent 2100 Bioanalyzer using 35- and 10380-bp markers. **(B)** cfDNA fragments lengths were shown for lupus nephritis (LN) individuals (n=3, gray) and non-LN individuals (n=6, blue). **(C)** Linear correlations between the cfDNA concentration and the fragmentation index (*r* = 0.4954, *P*< 0.0001). **(D)** The fragmentation index in the LN group was similar to that of the non-LN group (6 ([3-14] vs 14 [4-15]; *P* = 0.0794). **(E)** The mean of the Motif scores were similar in the two groups (0.0190 vs 0.0182; *P* =0.9048). **(F)** The mean of the DELFI scores in the LN group was significantly higher than that in the non-LN group (0.8970 vs 0.7632; *P* = 0.0238).

Recently, there has been much research interest in fragment omics, which is one of the molecular characteristics of cfDNA in plasma. **Figures 2A, B** show the overall size distribution of plasma cfDNA molecules. Most cfDNA fragments were enriched in the 153-198bp range. We described the fragmentation profile of plasma cfDNA using the number of electrophoretic peaks and the proportion of specific fragments. The fragmentation index of each sample was calculated on the basis of the number of electrophoretic peaks. Although the fragmentation index and cfDNA concentration, which were related to kidney involvement in patients with SLE, were positively correlated (**Figure 2C**), and the relationship between the fragmentation index and kidney involvement was unclear (**Figure 2D**). The motif and DELFI scores were the proportions of two different specific fragments. They were used to describe the fragmentation profile of the plasma cfDNA. We calculated the motif score and the DELFI score of the nine samples that underwent methylation sequencing separately (**Supplementary Table 6**). The mean motif scores in the LN and non-LN groups were similar (0.0190 vs. 0.0182; *P* = 0.9048; **Figure 2E**). The mean DELFI scores in the LN group were significantly higher than those in the non-LN group (0.8970 vs. 0.7632; *P* = 0.0238; **Figure 2F**). This means that patients with LN had shorter fragments of plasma cfDNA than those without LN.

### Different methylation level of plasma cfDNA in LN and non-LN

cfDNA methylation profiling was conducted at GeneCast Biotechnology Co. Ltd. using a capture-based next-generation-sequencing (NGS) panel designed primarily based on the publicly available TCGA dataset. A total of 8,975 DMPs were obtained by comparing the beta-values of TCGA cohort primary tumors and normal tissues, and 8,294 DMPs were obtained by comparing TCGA cohort primary tumor tissues and the peripheral blood samples of healthy individuals from GSE40279. The methylation panel was designed to cover the DMRs merged from these DMPs. The quality control data for methylation sequencing are shown in **Supplementary Table 7**.

We selected these features by comparing the methylation level of this cohort with the DMRs of the renal carcinoma data and the MCBs of the pan-cancer panel, except for kidney cancer. There were 68, 21, and one renal carcinoma-specific DMPs selected for KIRC, KIBP, and KICH, respectively, with a mean beta-value difference >0.3 and false discover rate (FDR)<0.05. Among each DMP set, DMPs with distances<250bp from the others were merged into a DMR. This procedure produced 823, 831, and 899 tissue-specific DMRs for KIRC, KIRP, and KICH, respectively, along with four tumor-specific DMRs for KIRC and one for KIRP. The cfDNA samples were hybridized to a self-designed methylation panel covering approximately 1Mb of hypermethylated regions and 82,400 CpGs. Adjacent CpG sites were merged into 6042 MCBs based on the correlations of beta-values. By comparing the mean beta-value or the MFR value, 20, nine, two, and two features were selected in DMRs and 73, 66, eight, and 10 features were selected in MCBs, respectively, under the condition of *P*< 0.05 and *P*< 0.01 (**Supplementary Table 8**).

**Figure 3:**
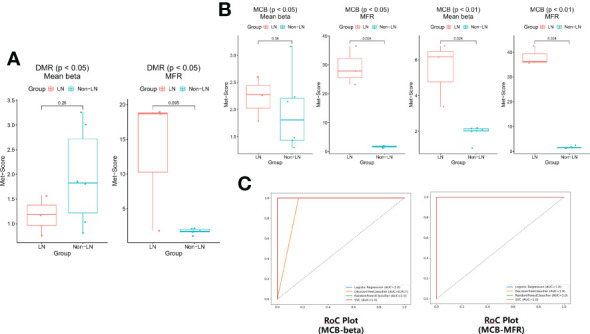
**(A)** The methylation scores were similar based on the DMRs of the renal carcinoma data in the two groups (*P* > 0.05). **(B)** The differences of methylation scores based on the MCBs of the pan-cancer panel except for kidney cancer between LN and non-LN (*P*< 0.05). **(C)** The leave-one-out cross-validation was conducted (logistic regression, decision tree classifier, random forest classifier, and SVC) and the models worked remarkably well.

The methylation scores of these features in each sample were calculated (**Supplementary Tables 9** and **10**). The entire flow of analysis is presented in **Supplementary Figure 1**. The mean methylation scores of the 20 and nine features selected in the DMRs were similar between the two groups (*P* > 0.05; **Figure 3A**). The number of features selected in DMRs under the condition of *P*< 0.01 was too small to count the methylation score. The mean methylation scores of the 73 features selected in MCBs by comparing the mean beta-value under the condition of *P*< 0.05 also were similar between the two groups (*P* > 0.05; **Figure 3B**). The mean methylation scores of the other 66, eight, and 10 features selected in the MCBs were significantly higher in the LN group than in the non-LN group (*P*< 0.05; **Figure 3B**).

To prevent overfitting caused by the excessive number of features, the eight and 10 features selected in MCBs by comparing the mean beta-value and the MFR value under the condition of *P*< 0.01 were modeled by machine learning. Using a threshold of 0.63 (logistic regression), 0 (decision tree), 0.23 (random forest) or 0.2 (SCV), P1, P2, and P3 were predicted as LN by each model based on beta-value (**Supplementary Table 11**). Using a threshold of 0.63 (logistic regression), 0 (decision tree), 0.23 (random forest) or 0.24 (SCV), P1, P2, and P3 were predicted as LN by each model based on the MFR value (**Supplementary Table 12**). The binary classification results were concordant among the four models. These results showed that the models worked remarkably well and that the features could distinguish LN from non-LN well (**Figure 3C**). The methylation levels of plasma cfDNA in patients with LN differed from those in patients without LN.

## Discussion

Previous studies (9–14, 17, 18) have noted the relationship between plasma cfDNA and disease severity in SLE (including renal involvement) and proposed that cfDNA might be a diagnostic and prognostic biomarker for SLE. The biological characteristics of cfDNA have been extensively discussed in previous SLE studies. Meanwhile, the exploration of cfDNA profiling in patients with SLE with renal involvement is still in its infancy. In this study, three different characteristics of plasma cfDNA from the perspectives of serology, fragment omics, and epigenetics were compared in patients with and without LN. We found lower concentrations and shorter fragments of plasma cfDNA in patients with LN than in those without LN and differences in methylation levels between the two groups.

Some studies (9–14) have confirmed that plasma cfDNA concentration is associated with disease severity in SLE. However, few studies (17, 18) have analyzed cfDNA concentration in LN. In this study of a cohort of 127 SLE patients (64 LN and 63 non-LN), the concentration of cfDNA in LN patients was significantly lower than that in non-LN patients. In addition, we also found that the widespread presence of anti-dsDNA antibodies in patients with LN might be one of the reasons for the reduction in cfDNA concentration, because the higher the titer of the anti-dsDNA antibody, the lower the concentration of cfDNA. A negative correlation between anti-dsDNA antibody levels and cfDNA concentration has been proposed in previous studies (9, 27). Since the deposition of immune complexes in the renal parenchyma is a key trigger of LN, the presence of anti-dsDNA antibodies is closely associated with renal involvement in patients with SLE (28–31). We found the proportion of patients with anti-dsDNA antibodies was significantly higher in the LN group (**Table 1**). A large number of anti-dsDNA antibodies combine with cfDNA in plasma to form immune complexes that are deposited into the kidney, leading to the production of multiple proinflammatory cytokines and chemokines (32). It may aggravate renal damage in patients with SLE based on the importance of chemokines in the pathogenesis of LN (33). At the same time, the formation of cfDNA/anti-dsDNA antibody immune complexes directly interferes with the detection of cfDNA or accelerates antibody-bound DNA elimination from circulation. Therefore, the presence of anti-dsDNA antibodies and their binding to circulating cfDNA may be responsible for the decrease in cfDNA concentrations in patients with LN.

Fragment omics of cfDNA has attracted increasing attention as a molecular characteristic of cfDNA (34). Plasma DNA fragments showed a characteristic size distribution, with the major peak of 166 base pairs and the smaller peaks occurring at 10 base pair intervals (35), suggesting that the fragmentation of cfDNA was a non-random process and might be related to the onset of SLE. In this study, we used three different methods to explore the fragmentation profile of plasma cfDNA. These results show that the LN group had a higher mean DELFI score for cfDNA, that is, a greater number of short fragments (100-150bp) of cfDNA. Lo et al. (14) found that the plasma DNA of patients with active SLE exhibited skewed molecular size distribution profiles, with a significantly increased proportion of short DNA fragments. The extent of plasma DNA shortening was correlated with the SLEDAI and anti-dsDNA antibody levels, and the short DNA fragments were more readily bound to immunoglobulin G in plasma (14). Zhang et al. (17) also observed these specific fragments in SLE samples, which were associated with dsDNA antibody titers, disease activity, and decreased kidney function. Short cfDNA fragments may have a stronger affinity for anti-dsDNA antibodies. More anti-dsDNA antibodies and a large number of short cfDNA fragments with a high affinity for anti-dsDNA antibodies are conditions for the formation of more immune complexes. The formation of the cfDNA/anti-dsDNA antibody immune complex could directly interfere with the detection of cfDNA or accelerate antibody-bound DNA elimination from circulation. In SLE patients with renal involvement, there is more opportunity for the coexistence of anti-dsDNA antibodies and short cfDNA fragments. This might explain the lower cfDNA concentration in patients with SLE with renal involvement from a fragmentomic perspective.

Increasing evidence suggests that epigenetic modifications, especially DNA methylation, play a critical role in the pathogenesis of SLE (2, 36, 37). Many differentially methylated CpG sites have been observed in SLE patients with renal involvement (38, 39). No studies have reported the methylation profiles of plasma cfDNA in patients with LN. In a series of studies by Lo et al. (14), shorter plasma DNA fragments and IgG-bound plasma DNA fragments tended to be more hypomethylated, and the extent of hypomethylation correlated with the SLEDAI score and anti-dsDNA antibody level. In this study, we calculated the methylation scores of cfDNA based on the MCB data of the pan-cancer panel using targeted methylation sequencing. The mean methylation score was higher in the LN group. The results were verified using a machine learning method. From a data perspective, since each MCB feature was a consolidation and arithmetic mean of multiple CpG islands, the practice reduced model variance due to overfitting during feature selection, and hence improved the robustness of the models. As our results were calculated based on cancer data, they were only theoretical and not representative of the entire population with LN. The targeted methylation sequencing was performed in this study instead of the Whole Genome Bisulfite Sequencing, which may result in some deviation between our results and the actual situation. Moreover, clinical studies often face real-world challenges in a limited cohort size, and the outcomes have unsurprisingly demonstrated a high level of variance across different platforms and studies. Most previous studies used the Illumina Methylation 450K array, while we used the NGS. DNA methylation is altered in tissue-specific and cell type or cell composition-specific manner (40). Many studies (41–45) suggest that DNA hypomethylation contributes to the autoimmunity of SLE, but the studied DNA is always derived from peripheral blood mononuclear cells or tissues, rather than cfDNA. Meanwhile, some studies show that DNA hypermethylation is associated with some immune-related signaling pathways (e.g., Wnt (46), TGF-beta (47)). The role of abnormal increases or decreases in DNA methylation in the pathogenesis of SLE or LN remains unclear. The most important implication of our results is the difference in cfDNA methylation levels between patients with LN and those without LN, which offers a promising platform for the non-invasive investigation of genomic epigenetic alterations that are specific to LN. Further studies are needed to fully elucidate the contribution of DNA methylation to disease heterogeneity.

To date, renal biopsy remains the most accurate standard for diagnosing the pathological pattern of LN, although it is invasive and not easily repeatable. Urine examination is an alternative method for rapid diagnosis of LN in clinical practice. However, urine examination cannot reflect the pathological pattern of kidney damage and might result in errors due to improper specimen collection. Therefore, there is an urgent need for a non-invasive, accurate, and easily repeatable biomarker for diagnosing the pathological pattern of LN. Our results support replacing the existing renal tissue biopsy with liquid biopsy of cfDNA for the diagnosis and molecular typing of LN was promising research.

This study had some limitations. Pathological classification is important for the diagnosis, treatment, and prognosis of LN. Unfortunately, no information on the pathological types of LN was collected from the cohort in this study, so the relationships between cfDNA biological characteristics and pathological types in patients with LN could not be further analyzed. Targeted methylation sequencing was performed rather than genome-wide methylation sequencing, which had adverse effects on the accuracy of the results. The results of our study need to be further validated in a larger and more diverse patient population.

The application of NGS, together with advanced computational methods, has greatly improved our understanding of cfDNA profiling in LN. Our study is probably the first to combine the serological, fragmentomic, and epigenetic characteristics of cfDNA to garner a more comprehensive exploration of cfDNA in patients with LN. Significant differences in the concentration, fragmentation profile, and methylation level of cfDNA between the LN and non-LN groups were observed in our study. Our study provides a basis for the feasibility of cfDNA based liquid biopsy as a replacement for renal tissue biopsies.

## Data availability statement

The datasets presented in this study can be found online at: https://www.ncbi.nlm.nih.gov/sra/PRJNA874316.

## Ethics statement

The studies involving human participants were reviewed and approved by Ethics Committee of the Second Affiliated Hospital of Fujian Medical University. The patients/participants provided their written informed consent to participate in this study.

## Author contributions

FW, Z-HP, and ZC designed the study. FW and H-BM collected samples, performed research, and wrote the manuscript. FW and Z-HP analyzed the data. All authors contributed to the article and approved the submitted version.
