# Supplementary material for: Serological, fragmentomic, and epigenetic characteristics of cell-free DNA in patients with lupus nephritis
Source: Front Immunol. 2022 Dec 12;13:1001690. doi: 10.3389/fimmu.2022.1001690 (PMC9791112; doi:10.3389/fimmu.2022.1001690)
Supplement: Supplementary file 1 [file DataSheet_1.zip › Supplementary_Material/Supplement Figure1.pdf]

(A)

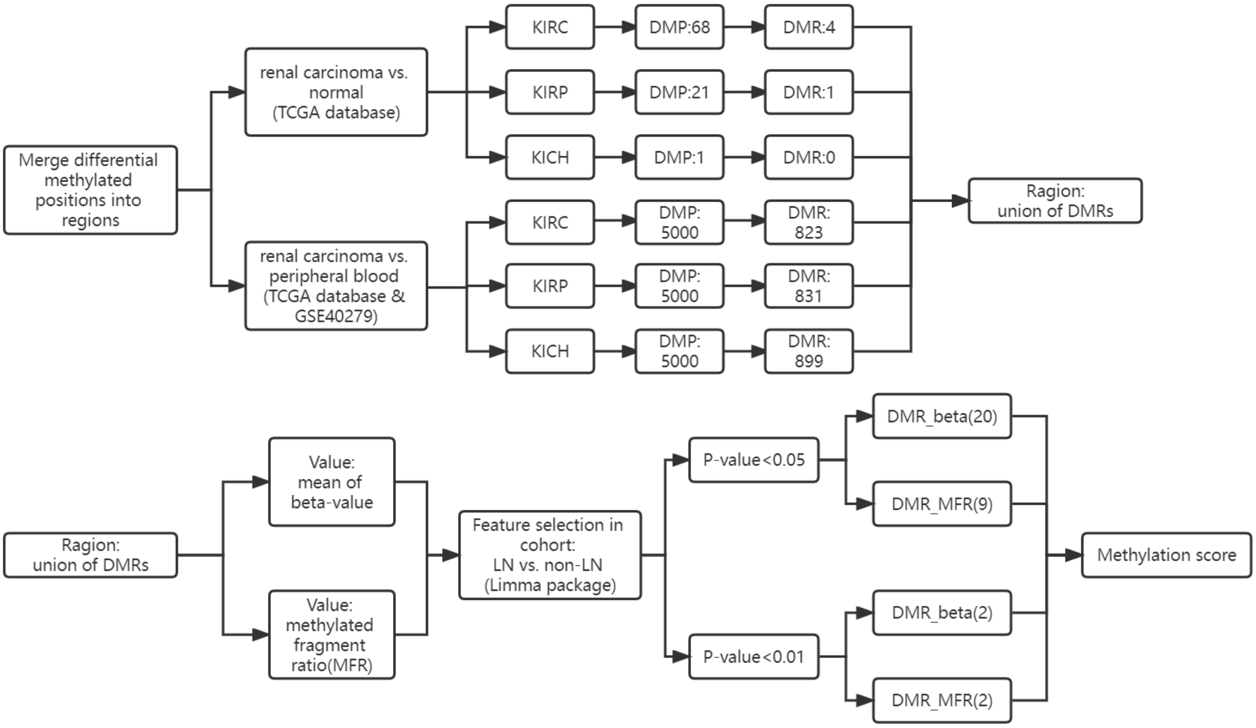

(B)

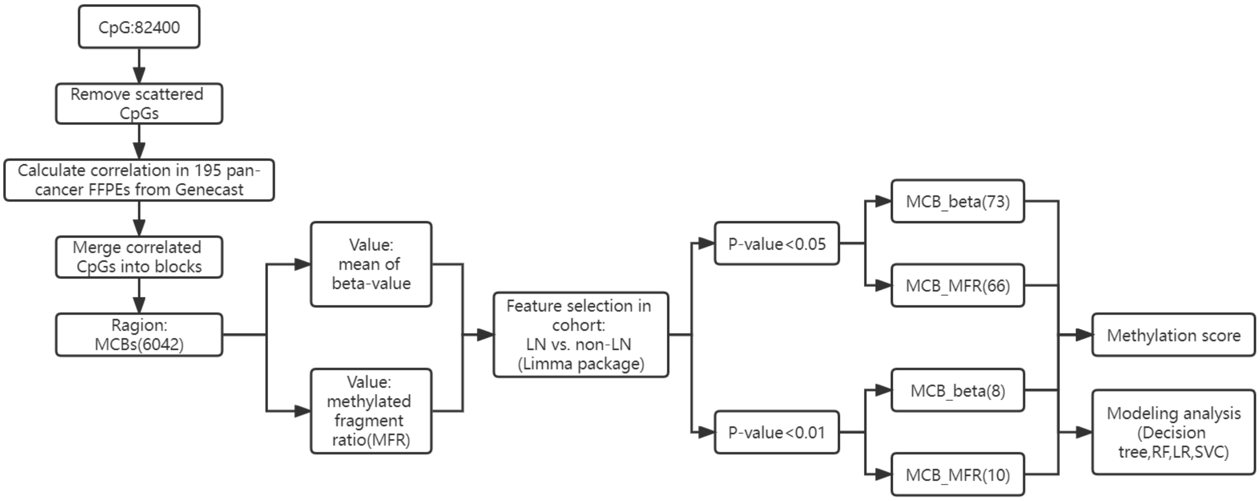

Supplement Figure 1. Methylation analysis flow charts based on the renal carcinoma data in TCGA database (A) and the pan-cancer panel (B).
