# Supplementary material for: Serological, fragmentomic, and epigenetic characteristics of cell-free DNA in patients with lupus nephritis
Source: Front Immunol. 2022 Dec 12;13:1001690. doi: 10.3389/fimmu.2022.1001690 (PMC9791112; doi:10.3389/fimmu.2022.1001690)
Supplement: Supplementary file 1 [file DataSheet_1.zip › Supplementary_Material/Supplementary Table 1.docx]

**Supplementary Table 1.** Patient demographics

|  | LN(n=3) | Non-LN(n=6) | *P*-Value |
| --- | --- | --- | --- |
| Age,years | 29(27.5-43) | 25.5(16-29) | NS |
| Gender,female,n(%) | 2(66.67) | 5(83.33) | NS |
| BMI，kg/m^2^ | 20.55(18.09-23) | 17.92(17.63-18.75) | NS |

n(%): number of patients (percent); NS: not significant; LN: lupus nephritis; BMI: body mass index.
