# Supplementary material for: Serological, fragmentomic, and epigenetic characteristics of cell-free DNA in patients with lupus nephritis
Source: Front Immunol. 2022 Dec 12;13:1001690. doi: 10.3389/fimmu.2022.1001690 (PMC9791112; doi:10.3389/fimmu.2022.1001690)
Supplement: Supplementary file 1 [file DataSheet_1.zip › Supplementary_Material/Supplementary Table 10.docx]

**Supplementary Table 10.** Methylation scores based on the MCB of pan-cancer panel

| **Patient** | **Group** | **MCB_beta**  **(P<0.05)** | **MCB_MFR**  **(P<0.05)** | **MCB_beta**  **(P<0.01)** | **MCB_MFR**  **(P<0.01)** |
| --- | --- | --- | --- | --- | --- |
| P1 | LN | 2.6176 | 27.8909 | 6.1714 | 36.2294 |
| P2 | LN | 1.7789 | 23.2361 | 3.3672 | 35.6160 |
| P3 | LN | 2.2774 | 36.5569 | 6.7826 | 42.5096 |
| P4 | Non-LN | 2.1365 | 1.9324 | 2.0862 | 1.7766 |
| P5 | Non-LN | 2.2307 | 1.5961 | 2.1949 | 1.3730 |
| P6 | Non-LN | 1.4168 | 1.1399 | 2.1635 | 1.2341 |
| P7 | Non-LN | 3.1708 | 1.9583 | 2.0303 | 2.5222 |
| P8 | Non-LN | 1.2889 | 1.7597 | 1.0496 | 1.4283 |
| P9 | Non-LN | 1.4847 | 1.5265 | 1.9979 | 1.4468 |
| mean_LN | | 2.2246 | 29.2280 | 5.4404 | 38.1184 |
| mean_non_LN | | 1.9547 | 1.6522 | 1.9204 | 1.6302 |
| Wilcox.test | | 0.381 | 0.024^*^ | 0.024^*^ | 0.024^*^ |

*P<0.05; MCB: methylation-correlated blocks; MFR: methylated fragment ratio; LN: lupus nephritis.
