# Supplementary material for: Serological, fragmentomic, and epigenetic characteristics of cell-free DNA in patients with lupus nephritis
Source: Front Immunol. 2022 Dec 12;13:1001690. doi: 10.3389/fimmu.2022.1001690 (PMC9791112; doi:10.3389/fimmu.2022.1001690)
Supplement: Supplementary file 1 [file DataSheet_1.zip › Supplementary_Material/Supplementary Table 6.docx]

**Supplementary Table 6.** Proportion of specific fragments of cell-free DNA

| Patient | Group | DELFI | Motif |
| --- | --- | --- | --- |
| P1 | LN | 0.9063 | 0.0194 |
| P2 | LN | 0.8963 | 0.0183 |
| P3 | LN | 0.8885 | 0.0194 |
| P4 | Non-LN | 0.8521 | 0.0183 |
| P5 | Non-LN | 0.8124 | 0.0189 |
| P6 | Non-LN | 0.7806 | 0.0198 |
| P7 | Non-LN | 0.6208 | 0.0123 |
| P8 | Non-LN | 0.8062 | 0.0206 |
| P9 | Non-LN | 0.7069 | 0.0190 |
| mean_LN |  | 0.8970 | 0.0190 |
| mean_non_LN |  | 0.7632 | 0.0182 |
| Wilcox.test |  | 0.0238^*^ | 0.9048 |

*P<0.05; DELFI: DNA evaluation of fragments for early interception; Motif: the frequency of each plasma DNA end motif; LN: lupus nephritis.
