# Supplementary material for: Serological, fragmentomic, and epigenetic characteristics of cell-free DNA in patients with lupus nephritis
Source: Front Immunol. 2022 Dec 12;13:1001690. doi: 10.3389/fimmu.2022.1001690 (PMC9791112; doi:10.3389/fimmu.2022.1001690)
Supplement: Supplementary file 1 [file DataSheet_1.zip › Supplementary_Material/Supplementary Table 7.docx]

**Supplementary Table 7.** Quality control data of methylation sequencing

| **Patient** | **Panel size**  **(M bp)** | **Depth**  **(x)** | **Amount of sequencing data (Gb)** |
| --- | --- | --- | --- |
| P1 | 0.998 | 973.19 | 7.64 |
| P2 | 0.998 | 1057.29 | 9.03 |
| P3 | 0.998 | 1001.55 | 8.16 |
| P4 | 0.998 | 922.17 | 7.40 |
| P5 | 0.998 | 899.28 | 8.24 |
| P6 | 0.998 | 945.53 | 7.29 |
| P7 | 0.998 | 534.04 | 4.56 |
| P8 | 0.998 | 909.19 | 7.31 |
| P9 | 0.998 | 894.20 | 6.88 |
