# Supplementary material for: Serological, fragmentomic, and epigenetic characteristics of cell-free DNA in patients with lupus nephritis
Source: Front Immunol. 2022 Dec 12;13:1001690. doi: 10.3389/fimmu.2022.1001690 (PMC9791112; doi:10.3389/fimmu.2022.1001690)
Supplement: Supplementary file 1 [file DataSheet_1.zip › Supplementary_Material/Supplementary Table 8.docx]

**Supplementary Table 8.** Selecting criteria and the number of features

| ***P*-value Cutoff** | **DMR** | | **MCB** | |
| --- | --- | --- | --- | --- |
|  | **beta-value** | **MFR** | **beta-value** | **MFR** |
| <=0.05 | 20 | 9 | 73 | 66 |
| <=0.01 | 2 | 2 | 8 | 10 |

DMR: different methylation region; MCB: methylation-correlated blocks; MFR: methylated fragment ratio.
