# Supplementary material for: Serological, fragmentomic, and epigenetic characteristics of cell-free DNA in patients with lupus nephritis
Source: Front Immunol. 2022 Dec 12;13:1001690. doi: 10.3389/fimmu.2022.1001690 (PMC9791112; doi:10.3389/fimmu.2022.1001690)
Supplement: Supplementary file 1 [file DataSheet_1.zip › Supplementary_Material/Supplementary Table 9.docx]

**Supplementary Table 9.** Methylation scores based on the DMRs of renal carcinoma

| **Patient** | **Group** | **DMR_beta**  **(P<0.05)** | **DMR_MFR (P<0.05)** | **DMR_beta^*^ (P<0.01)** | **DMR_MFR^*^ (P<0.01)** |
| --- | --- | --- | --- | --- | --- |
| P1 | LN | 1.1862 | 18.9435 | - | - |
| P2 | LN | 0.7497 | 1.8538 | - | - |
| P3 | LN | 1.5752 | 18.6894 | - | - |
| P4 | Non-LN | 1.8458 | 1.7352 | - | - |
| P5 | Non-LN | 3.0080 | 2.1346 | - | - |
| P6 | Non-LN | 0.7988 | 1.0681 | - | - |
| P7 | Non-LN | 3.2657 | 2.1754 | - | - |
| P8 | Non-LN | 1.0232 | 1.7932 | - | - |
| P9 | Non-LN | 1.8085 | 1.6644 | - | - |
| mean_LN | | 1.1704 | 13.1622 | - | - |
| mean_non_LN | | 1.9583 | 1.7618 | - | - |
| Wilcox.test | | 0.262 | 0.095 | - | - |

DMR: different methylation region; MFR: methylated fragment ratio; LN: lupus nephritis; *: The calculation failed because the number of features was insufficient.
